# University social responsibility under the influence of societal changes: Students’ satisfaction and quality of services in Saudi Arabia

**DOI:** 10.3389/fpsyg.2022.976192

**Published:** 2022-09-06

**Authors:** Abdulelah A. Alghamdi

**Affiliations:** Faculty of Education, Umm Al-Qura University, Makkah, Saudi Arabia

**Keywords:** university social responsibility, students’ satisfaction, quality of services, societal changes, Saudi Vision 2030

## Abstract

Universities contribute to shaping the identity of a nation with their major university social responsibility (USR) in addition to their academic purposes and corporate strategies. In Saudi Arabia with Vision 2030, universities are facing a transformation in adapting to the societal changes and implementing a socially responsible management, considering the satisfaction of their most important stakeholders (i.e., the students) and the quality of services offered to them. This study aims to explore how USR fulfills the societal changes in Saudi Arabia from the perspective of university students in addition to inspecting USR’s relationships with the students’ satisfaction and the quality of services offered to them. A self-report study was conducted with 350 undergraduate students in the Faculty of Social Sciences in a Social Work program at a University in the Western region of Saudi Arabia. An inside–outside (I–O) map of USR was applied to investigate the relationships among USR’s aspects. The study results demonstrated a moderate level of agreement by students toward the university’s fulfillment of its USR, quality of services offered to them, and their satisfaction. In contrast, the results of a regression analysis revealed that all USR aspects could explain only 13% of the students’ satisfaction. Legal responsibilities, among all USR aspects, exhibited the highest influence on students’ satisfaction. Gender differences existed in favor of female students’ perceptions toward the university’s fulfillment of its USR. The I–O map provided interesting insights to interpret the correlations among all USR aspects under the influence of societal changes that have occurred under Saudi Vision 2030.

## Introduction

As universities have adapted themselves to a significant change process to pursue the approach and purpose of education in the context of globalization, they have currently become an important icon in achieving the demands of society (Chicharro and Carrillo, 2009). Understanding the consequences of social changes occurring in current societies requires universities to be open-minded to new challenges and opportunities related to the achievement of their academic purposes, which coincide with the needs of these societies (Gaete Quezada, 2015).

Therefore, universities contribute to shaping the identity of a nation with their major responsibilities in addition to providing educational services. Thereby, universities play a crucial role in the incorporation of social responsibility in their academic purposes, mission, vision, and corporate strategy (Sullivan, 2003; Muijen, 2004). In Saudi Arabia, the societal changes that were driven by Saudi Vision 2030, which is a unique transformative economic and social reform blueprint that is opening Saudi Arabia up to the world, enriching the lives of Saudi citizens and fulfilling the Kingdom’s vast potential, universities are experiencing transformation to meet the needs and ambitions of society (Saudi Vision 2030, 2016). This is represented in a change process that has led to establishing degrees, diplomas, and curricula that provide a suitable environment for Saudi universities to implement socially responsible management, considering their stakeholders’ requirements and satisfaction (Al-Ahmadi, 2016). From this perspective, university social responsibility (USR) guides this change of university culture to a culture of responsibility considering the satisfaction of a university’s most important stakeholders: its students (Sánchez-Hernández and Mainardes, 2016).

Universities are increasingly concentrating on meeting their students’ needs and expectations (DeShields et al., 2005). Given that universities are currently experiencing a significant change in their approaches and services of education while facing a competitive environment in a globalizing context, they have applied strategies to address their quality of services as an antecedent of their students’ satisfaction (Vázquez et al., 2016). One of these strategies is measuring USR as a determining factor and the main reason behind student satisfaction in addition to the quality of services (Sánchez-Hernández and Mainardes, 2016; Vázquez et al., 2016). In this context, analyzing students’ perceptions as a university’s most important stakeholders with the societal changes process that has resulted from Vision 2030 in Saudi universities is important for measuring USR as an index of student satisfaction and the quality of services provided by universities. Thus, this study aims to identify how USR helps in meeting the requirements of societal changes in Saudi Arabia based on student perceptions, in addition to inspecting USR’s relationship with student satisfaction and the quality of services offered to them.

## Literature review

### University social responsibility

Universities are one of society’s components that play a part in developing the community and raising socially acceptable behaviors of its members by connecting their social responsibility criteria in management, learning, and research with the emerging needs of society in the context of globalization (Latif, 2018). This requires universities to reformulate their strategies, especially with the increasingly competitive market of higher education and the existing diverse challenges and obstacles (Burcea and Marinescu, 2011; Vázquez et al., 2014). As a result, a university’s performance assessment should reflect its commitment to ensuring the provision of fair and transparent operating systems and increasing the positive influence of its stakeholders on the environment by inculcating a good model of ethical behavior that is implemented in real life (Gomez, 2014). Universities are thus not only educational service providers but also organizations that produce trained citizens in terms of competency in satisfying the needs of their stakeholders, thereby imposing greater social responsibility on them (Myers, 2002; Sullivan, 2003; Wilhite and Silver, 2005; Kunstler, 2006).

From this perspective, it is reasonable to define USR as “a concept whereby a university integrates all of its functions and activities with the society needs through active engagement with its communities in an ethical and transparent manner which is aimed to meet all stakeholders’ expectations” (Esfijani et al., 2013). This means that USR provides educational services and spreads knowledge ethically with good management, as well as encourages a university’s stakeholders to enhance sustainable development in their society by raising their sense of responsible citizenship by adopting views and values of the university (Vázquez et al., 2015). In this manner, universities can thrive in the competitive higher education market by incorporating social responsibility into their vision and strategies (Jimena, 2011).

Some academics have studied the social responsibility of universities by integrating it with the proposed strategies in universities (Muijen, 2004), investigating its relationship with the academic achievement of teachers (Junyi et al., 2011), and highlighting its normative concepts, origins, importance, and implementation practices in the university (Gomez, 2014). Others have concentrated on identifying the implications of USR on the design of university marketing strategies (Vázquez et al., 2014), measuring the impacts of USR with selected indicators on society from the perspective of business students (Sánchez-Hernández and Mainardes, 2016), and developing a valid scale to measure USR through wider social responsibility dimensions (Latif, 2018).

However, social responsibility is not the same in every sector of society and for all stakeholders (Sallyanne Decker, 2004; Latif, 2018). As universities impact their respective societies, it is important that they take responsibility for the individual impact of their strategies, policies, and performances (Argandoña, 2012; Vázquez et al., 2014). Likewise, social responsibility within universities is not the same for all stakeholders (Garde Sánchez et al., 2013). Verdeyen et al. (2004) classified stakeholders into internal and external stakeholders on the basis of their location inside and outside the organization. In a university setting, internal stakeholders include students who are influenced by initiatives and social responsibility of the university (Latif, 2018), and put a greater emphasis on their expectations and needs to be met considering them as the most important stakeholders (DeShields et al., 2005; Sánchez-Hernández and Mainardes, 2016). From this viewpoint, in the current study, students’ perceptions, and experience related to USR represent a valuable input for divulging the fulfillment of the university toward them.

In the context of implementing USR in Saudi Arabia, Vision 2030 has shaped the roles of Saudi universities in society’s future improvement. Vision 2030 is built around three main themes: a vibrant society, a thriving economy, and an ambitious nation. Regarding the second theme, Vision 2030 asserts that a thriving economy should provide opportunities for all stakeholders by building an educational system that aligns with society’s needs and contributes to its economic growth. In higher education, universities need to meet the requirements of the job market and increase student awareness regarding educational pathways and future career decisions. This vision aims to position at least five Saudi universities among the top 200 universities internationally to help students achieve above average results in terms of global education indicators. This aim is achievable through creation and implementation of a modern curriculum that focuses on rigorous standards in skills and personal development, publishing a sophisticated range of education outcomes, investing in strategic partnerships with apprenticeship providers from large private companies, and developing job specifications for each academic field (Saudi Vision 2030, 2016).

Based on these considerations, universities are responsible for improving their education planning, monitoring, evaluation, and outcomes, as well as their effects on stakeholders and society (Argandoña, 2012; Vázquez et al., 2014).

### Quality of university services and students’ satisfaction

Saudi Arabia’s Vision 2030 falls within the premise of Saudi universities to promote their social responsibilities and rethink their impacts on the knowledge, values, and behaviors of stakeholders, including students. This highlights the role of universities in terms of provision of quality services and commitment to meeting student requirements regarding their future, which influences their level of satisfaction.

The importance of student satisfaction is driven by their position as the main stakeholders of educational services and in turn, its positive relationship with the aspects of academic performance, motivation, and retention within universities (Elliott and Shin, 2002; Lee and Tai, 2008). Being responsive to student demands and expectations means that the university needs to seek student satisfaction to reflect positively on their performance and achieve high outcomes. From this perspective, student satisfaction can be defined as a short-term attitude resulting from the evaluation of an actual performance that meets or exceeds students’ expectations (Elliott and Shin, 2002). Therefore, student satisfaction is a vital factor for Saudi universities to fulfill the requirements of Vision 2030.

Meanwhile, quality assurance is another important aspect of Saudi Arabia’s Vision 2030. This includes the quality of services concept, which is important in different sectors, particularly in the higher education sector (Saudi Vision 2030, 2016). University education associates with quality in order to respond to building an education system that fulfills the social responsibilities of universities and works in alignment with society’s needs. In this context, the quality of university services can be defined as “the difference between what a student expects to receive and his/her perceptions of actual delivery” (O’Neill and Palmer, 2004). Service quality in higher education has recently been studied globally (Barnes, 2007; Pereda et al., 2007; Angell et al., 2008; Sohail and Hasan, 2021). Regarding Saudi Arabia’s Vision 2030, universities should raise the social responsibilities of students toward their society and become an organization based on the quality of services provided in the higher education sector to contribute to the development and growth of other sectors in Saudi Arabia (Saudi Vision 2030, 2016).

### Research questions

Given the above literature review, the societal changes in Saudi Arabia that are driven by Vision 2030 require universities to be socially responsible to guide these changes in addition to the quality of services provided to satisfy their students as the main stakeholders. This is to ensure that these students can contribute to the growth of society, and fulfill the goals of Saudi Vision 2030. From this perspective, the following research questions were formulated:

*Q1: How can USR fulfill the societal changes, driven by Saudi Vision 2030, from the perspective of university students?*

*Q2: How are the relationships among USRs in Saudi Arabia perceived in terms of (a) the satisfaction of students and (b) the quality of services offered to them?*

In this study, gender was an important consideration for further research in this context, because of the segregation in Saudi educational institutions. Accordingly, the following research questions were formulated:

*Q3: How can USR fulfill the societal changes, driven by Saudi Vision 2030, in Saudi Arabia from the perspective of male and female university students?*

*Q4: How are the relationships among USRs in Saudi Arabia perceived in terms of (a) the satisfaction of male and female students and (b) the quality of services offered to both of them?*

## Methodology

### Procedure

Due to the importance of awareness of the concept of USR among the study sample, self-reported data were collected through a survey addressed to university students studying in a respected Saudi university in the Faculty of Social Sciences in a Social Work program. To validate the survey results, the Arabic version of the content of the scales adopted in this study was reviewed by Saudi experts on the research subject. After receiving consensus on the survey’s validity, a pilot study was conducted with a group of 25 university students to obtain feedback. A factor analysis (principal component analysis [PCA]) was conducted using the Kaiser-Meyer-Olkin (KMO) test and Bartlett’s test of sphericity (BTS) to ensure that the items of each scale measured one representative factor. The entire content of the survey was available online as Supplementary Information (see Supplementary Data Sheet 1). After obtaining ethics approval (Number: 4301054743), 438 emails were sent randomly to university students studying Social Work during the second semester of 2019/2020. Before the survey was initiated, all participating students provided anonymous consent. Approximately 359 responses were obtained (i.e., 81.9% response rate), of which 350 responses were complete and valid.

### Measures

The scales’ items were built based on the available literature. The original English version of the survey was subjected to two-way translation to ensure the translation quality of the scales and their meaning equivalence. The survey comprised four sections to collect data related to the participants’ socio-demographic details (i.e., their age and gender) and their perceptions toward the university’s fulfillment of its social responsibilities, with investigation of their relationships to the participants’ satisfaction and the quality of services offered to them (see Supplementary Appendix 1). The participants socio-demographic details were in the first section.

The second and third sections included the USR scales. The current study aimed to measure Saudi USR from the perspective of university students by building a scale based on the following two aspects: stakeholders’ perceptions and the university’s organizational structure. In this context, academic studies on how universities implement their social responsibility principles while simultaneously considering both these aspects are lacking, and most of the aforementioned studies have considered these aspects separately (Latif, 2018). The fact that the USRs within universities are not the same for all stakeholders (Garde Sánchez et al., 2013) and not the same in each society due to the different effects of the universities’ strategies and performance (Argandoña, 2012), indicates that universities have to take their responsibilities externally to the surrounding society and internally within the university, just like any other organization (Macassa et al., 2017).

From this perspective and based on existing literature (Sánchez-Hernández and Mainardes, 2016; Vázquez et al., 2016; Latif, 2018), the USR scale was built to measure the internal and external social responsibilities of universities in Saudi Arabia based on students’ perceptions as the main stakeholders of universities. Internal responsibilities included operational and legal responsibilities, while external responsibilities included voluntary and community responsibilities. Thus, the USR scale was divided into four subscales: operational responsibility scale (includes seven items), legal responsibility scale (includes seven items), voluntary responsibility scale (includes five items), and community responsibility scale (includes six items). These scales used a five-point Likert scale ranging from 1 (strongly disagree) to 5 (strongly agree).

In the fourth and fifth sections, the scale of life satisfaction [developed by Vázquez et al. (2016)], which includes six items, and the scale of quality of services [developed by Vázquez et al. (2015)], which includes five items, were adopted to investigate the relationships between students’ perceptions in terms of the university fulfilling its internal and external social responsibilities and their satisfaction based on the quality of services offered to them. These scales used a five-point Likert scale ranging from 1 (strongly disagree) to 5 (strongly agree).

### Strategy for mapping university social responsibility

Considering that universities can burgeon in the competitive higher education market by focusing on their social responsibilities in their vision and strategies (Jimena, 2011), resulting in performing their roles toward enhancing sustainable development in society and satisfying their stakeholders inside and outside of the universities. From this perspective, the social responsibilities within universities have been extensively studied to build a foundation and framework for understanding the nature of these responsibilities (Sánchez-Hernández and Mainardes, 2016; Vázquez et al., 2016; Latif, 2018). However, most of these studies have not proposed a unified strategy for conceptualizing the USR framework, because social responsibilities are not the same for all stakeholders and all societies.

Striking the right balance between the social responsibilities inside and outside of a university is critical as the influence of a university’s commitment to its social responsibilities is not limited to how its status is perceived but how much it influences and benefits the stakeholders and society. Nevertheless, universities have lacked the strategies required to get the balance right.

To address this issue, a strategy called the inside–outside (I–O) map of USR was developed. This strategy enables universities to display their USR on a perceptual map on the basis of both internal responsibilities, which serve the inside community, and external responsibilities, which serve the outside community. This strategy can assist a university as an organization to directly inspect its social responsibilities both internally and externally based on its principles and stakeholders’ perceptions. This allows each university to satisfy its stakeholders and determine the underlying factors that manipulate its responsibilities. In the developed I–O map, internal and external social responsibilities are not contradictory aims; universities can choose to pursue both and maximize their influence.

By considering the proposed framework of I–O map of USR for various social responsibilities internally and externally, the USRs along with the expected correlations among them can be divided into four quadrants (see Supplementary Appendix 2).

Social responsibilities with high correlations (SRHC), located in the upper-right quadrant, are correlated strongly and act toward achieving great value for the inside and outside communities. Social responsibilities toward the outside community (SROC), which have a major impact on the external environment, are located in the lower-right quadrant. These responsibilities, which are aimed at increasing the value of a university for society, are a competitive aspect within the higher education market under the societal changes occurring locally or even globally. Social responsibilities with low correlations (SRLC) are weakly correlated and do not fit properly to serve the targets of a university toward the inside and outside communities. These responsibilities are located in the lower-left quadrant. Despite their low correlation and minor group of influenced communities, they play an important role in implementing the principles of university responsibilities separately. In the upper-left quadrant are social responsibilities toward inside community (SRIC), which play an important role in increasing the satisfaction of the inside community and its loyalty toward the success of university strategies and polices.

Through these four quadrants of the I–O map of USR, visualization of USRs can assist universities address and follow their social responsibilities effectively. In the current study, USRs are represented in the form of an I–O map based on the perceptions of the main university stakeholders (i.e., the students). The developed map links student perceptions about USRs with their influences. The working mechanism of this map is represented in placing USR according to the correlation’s score among the responsibilities in each quadrant with bubbles displayed and sized according to the correlation score based on student perceptions in different USR aspects (i.e., operational, legal, voluntary, and community responsibilities). Each quadrant carries different correlations for each USR aspect. The distribution of the responsibilities’ correlations across the map offers insights into different added values and influences. In the current study, the I-O map with USR’s correlations is depicted in the discussion section.

### Data analysis

The quantitative data obtained from the surveys were analyzed using the software package SPSS. PCA was conducted on the scales of the survey, using the KMO test and BTS, to ensure that items of each scale gauged one representative factor. A descriptive analysis was conducted to gauge the categorical variables’ frequencies and to determine the means and standard deviations of each scale. Independent-samples *t*-tests were applied to identify the differences between the USR scores for male and female students, while Pearson correlation coefficients were utilized to explore the correlations among the USR aspects, quality of university’s services (QUS), and university students’ satisfaction (USS). Finally, a sample and a multilabel regression analyses were performed to predict QUS and USS. Parametric tests were considered appropriate as both the sample size and data met the requirements for parametric testing.

## Findings

### Participant characteristics

The demographic information of the sample is displayed in Table 1. A total of 350 students, both men and women, from a University in the Western region of Saudi Arabia participated in this study; these students were pursuing a bachelor’s degree in the Social Work program. The proportion of women (51.4%) was slightly more than that of men (48.6%), with most participants aged between 19 and 23 years.

**TABLE 1:** Demographic information for the sample.

		Number of participants	Percentage
Age	19–23	287	82%
	24 and more	63	18%
Gender	Men	170	48.6%
	Women	180	51.4%

### Students’ perceptions of university social responsibility aspects

In this study, student perceptions of USR were measured through 25 statements divided into two scales (internal and external social responsibilities of the university), which included four sub-scales representing the four USR aspects (operational, legal, voluntary, and community responsibilities). To validate these scales and sub-scales, they were subjected to PCA. Prior to performing PCA, the suitability of the data for factor analysis was assessed. By inspecting the correlation matrix, many coefficients with values of 0.3 and above were identified. In each scale and sub-scale, the KMO value was 0.85, which exceeded the recommended value of 0.6, and BTS value reached statistical significance (*p* = 0.000), supporting the factorability of the correlation matrix. PCA detected the presence of only one factor in each scale and sub-scale with eigenvalue (λ) exceeding 1.0. Reliability was assessed with Cronbach’s alpha (α), which showed a high level of reliability for the items of each scale exceeding the value of 0.8. This information is presented in Table 2.

**TABLE 2:** Total variance explained by each factor of USR’s aspects.

N*	Factors	Factor loading	λ	% variance	α
Internal social responsibilities of university			**7.391**	**52.792**	**0.93**
	*Operational responsibilities*		3.934	56.204	0.87
1	Ensuring the existence of an appropriate study environment for all students	0.831			
2	Commitment to providing equal and diverse communication channels for students	0.814			
3	Working continuously to develop condition of the educational environment	0.769			
4	Undertaking numerous initiatives to improve the environment	0.765			
5	Promoting freedom of expression, dialogue, and debate	0.719			
6	Providing all required sources of knowledge	0.684			
7	Encouraging engagement in activities that help develop knowledge, skills, and behavior	0.648			
	*Legal responsibilities*		4.108	58.685	0.88
1	Performing all legal duties for students	0.840			
2	The existence of honesty, transparency, and integrity in all transactions	0.821			
3	Complying with the general rules and regulations	0.816			
4	Respecting student rights and treating them fairly and without discrimination	0.805			
5	Commitment to implementing regulations of behavior and activity	0.763			
6	Clear procedures for reporting in case of violations	0.657			
7	Working in accordance with the values, principles, and customs of society	0.633			
External social responsibilities of university			**6.459**	**58.714**	**0.93**
	*Voluntary responsibilities*		3.122	62.443	0.84
1	Contributing to voluntary activities within the community	0.823			
2	Undertaking steps that help prevent environmental pollution	0.819			
3	Providing opportunities within the community for volunteer students to expand their expertise	0.816			
4	Encouraging initiatives of students toward preserving the environment	0.802			
5	Providing financial support for extracurricular activities	0.682			
	*Community responsibilities*		3.895	64.909	0.89
1	Supporting partnerships with the private sector to develop required students’ skills	0.844			
2	Providing university community employment opportunities	0.833			
3	Understanding the needs of community and working in consultation whenever possible	0.812			
4	Educating students about their social responsibility in their specializations	0.789			
5	Supporting social and economic research that impacts society	0.783			
6	Supporting and working with associations in line with the university’s mission in society	0.770			

Kaiser-Meyer-Olkin measure of sampling adequacy = 0.946, Bartlett’s test of sphericity, p < 0.001.

* Order of items according to factor loading.

To determine whether the mean score of the scales for each item could be described as low, medium, or high in the descriptive statistics for the perceptions of university students toward the four aspects of USR (i.e., operational, legal, voluntary, and community responsibilities), QUS, and USS, the following descriptors were applied to the survey results. A mean below two represented a low level of agreement, a mean between 2.1 and 3.5 represented a moderate level of agreement, and a mean above 3.6 represented a high level of agreement.

The results obtained from student perceptions in relation to the four aspects of USR, as measured through the 25 statements, showed moderate to low levels of agreement, with the overall mean ranging between 1.93 and 2.06 and the standard deviation ranging between 0.80 and 0.88. This indicates a perception by students of a moderate level of agreement by the university to fulfill its operational, legal, voluntary, and community responsibilities toward its students. In addition, the results showed a moderate level of agreement for QUS, with the overall mean of 2.88 and standard deviation of 0.89, as well as that for USS, with the overall mean of 2.49 and standard deviation of 0.93 (see Supplementary Appendix 3 for detailed results).

In terms of gender differences, the results of the *t*-test revealed that the variances for the two groups (men/women) were the same for the four USR aspects and for QUS, but not for USS. Furthermore, there were statistically significant differences in the mean scores of men’s and women’s perceptions for USS and the four aspects of USR, but not for QUS. However, the effect size obtained following Cohen’s (2013) guidelines to interpret the Eta squared values (η^2^) (0.01 = small effect, 0.06 = moderate effect, 0.14 = large effect) was very small according to the Eta squared values for all four aspects of USR and students’ satisfaction. The findings are presented in Table 3.

**TABLE 3:** Gender comparison for the factors of USR, QUS, and USS.

Factors	*Gender	Overview result		*t*-test for equality of means			*η^2^*
		Mean	St. Dev.	*t*	df	Sig. (two-tailed)	
**Operational responsibilities**							
	Man	1.81	±0.757	2.783	384	0.006	0.02
	Woman	2.05	±0.815				
**Legal responsibilities**							
	Man	1.85	±0.767	3.130	384	0.002	0.03
	Woman	2.13	±0.902				
**Voluntary responsibilities**							
	Man	1.87	±0.771	2.535	384	0.012	0.02
	Woman	2.09	±0.804				
**Community responsibilities**							
	Man	1.92	±0.861	2.945	384	0.003	0.02
	Woman	2.20	±0.888				
**QUS**							
	Man	2.86	±0.918	0.349	384	0.727	–
	Woman	2.90	±0.862				
**USS**							
	Man	2.29	±0.822	4.061	342.1	0.000	0.05
	Woman	2.68	±0.993				

*Number of participants: Man = 170, Woman = 180.

### Correlations among university social responsibility aspects, quality of university’s services, and university students’ satisfaction

The findings showed student perceptions of a moderate level of agreement in terms of the university fulfilling its four USR aspects. In addition, the findings showed a moderate level of QUS provided to students and their associated satisfaction, as presented in Supplementary Appendix 3. Considering these results, the Pearson correlation coefficient (PCC) was used to determine the nature of the relationships among the four USR aspects, QUS, and USS. The strength of the correlation was interpreted according to the guidelines of Cohen (2013), who suggested that a PCC value (*r)* ranging from 0.10 to 0.29 indicates a weak correlation, from 0.30 to 0.49 indicates a medium correlation, and that from 0.50 to 1.0 indicates a strong correlation.

#### University social responsibility aspects, quality of university’s services, and university students’ satisfaction

The results revealed that the correlations among all aspects of USR, QUS, and USS were positive. Moreover, a strong positive correlation existed among all four aspects of USR and between QUS and USS. However, weak positive correlations were found between each of QUS and USS and the aspects of voluntary and community responsibilities, whereas medium correlations were found between each of QUS and USS and the aspects of operational and legal responsibilities, as presented in Table 4.

**TABLE 4:** Pearson correlations among USR aspects, QUS, and USS.

	1	2	3	4	5	6	*M*	±SD
1. Operational responsibilities	–						1.93	±0.80
2. Legal responsibilities	0.85**	–					1.99	±0.85
3. Voluntary responsibilities	0.71**	0.64**	–				1.98	±0.79
4. Community responsibilities	0.73**	0.68**	0.85**	–			2.06	±0.88
5. QUS	0.33**	0.38**	0.28**	0.29**	–		2.88	±0.89
6. USS	0.33**	0.37**	0.24**	0.27**	0.61**	–	2.49	±0.93

** Correlation is significant at the 0.01 level (two-tailed).

#### Aspects of university social responsibility

As indicated in Table 5, the correlation matrix of USR variables showed a predominantly strong-to-medium positive correlation among all variables of operational and legal responsibilities and among all variables of voluntary and community responsibilities. In contrast, a predominantly medium positive correlation existed between the variables of operational and legal responsibilities on one hand and those of voluntary and community responsibilities on the other hand.

**TABLE 5:** Correlation matrix of USR’s variables.

	Q1	Q2	Q3	Q4	Q5	Q6	Q7	Q8	Q9	Q10	Q11	Q12	Q13	Q14	Q15	Q16	Q17	Q18	Q19	Q20	Q21	Q22	Q23	Q24	Q25
Q-O1	1																								
Q-O2	0.638	1																							
Q-O3	0.507	0.532	1																						
Q-L4	0.516	0.511	0.480	1																					
Q-O5	0.378	0.380	0.448	0.414	1																				
Q-O6	0.480	0.519	0.432	0.470	0.537	1																			
Q-O7	0.589	0.640	0.470	0.544	0.422	0.605	1																		
Q-L8	0.571	0.723	0.543	0.526	0.421	0.490	0.703	1																	
Q-O9	0.394	0.498	0.405	0.412	0.318	0.426	0.571	0.580	1																
Q-L10	0.447	0.551	0.472	0.429	0.439	0.454	0.585	0.595	0.495	1															
Q-L11	0.485	0.557	0.515	0.448	0.393	0.421	0.540	0.597	0.510	0.685	1														
Q-L12	0.371	0.430	0.384	0.409	0.365	0.386	0.442	0.457	0.430	0.593	0.653	1													
Q-L13	0.297	0.312	0.328	0.267	0.411	0.398	0.461	0.392	0.430	0.454	0.450	0.438	1												
Q-L14	0.518	0.601	0.495	0.499	0.402	0.460	0.621	0.697	0.558	0.561	0.603	0.518	0.479	1											
Q-V15	0.446	0.358	0.366	0.433	0.415	0.524	0.489	0.418	0.275	0.405	0.369	0.337	0.326	0.455	1										
Q-V16	0.507	0.497	0.417	0.471	0.372	0.522	0.544	0.515	0.352	0.388	0.371	0.284	0.241	0.482	0.671	1									
Q-C17	0.409	0.387	0.387	0.414	0.375	0.375	0.464	0.458	0.307	0.357	0.360	0.272	0.231	0.465	0.524	0.645	1								
Q-C18	0.501	0.592	0.516	0.449	0.406	0.498	0.557	0.553	0.405	0.467	0.496	0.371	0.276	0.541	0.504	0.539	0.616	1							
Q-C19	0.391	0.360	0.392	0.383	0.401	0.373	0.432	0.385	0.316	0.404	0.410	0.332	0.317	0.428	0.501	0.432	0.569	0.633	1						
Q-V20	0.430	0.479	0.398	0.427	0.317	0.343	0.472	0.464	0.410	0.346	0.383	0.272	0.281	0.454	0.384	0.496	0.495	0.623	0.505	1					
Q-V21	0.428	0.385	0.372	0.396	0.330	0.416	0.441	0.405	0.325	0.338	0.364	0.349	0.277	0.426	0.548	0.526	0.469	0.463	0.498	0.457	1				
Q-V22	0.456	0.414	0.459	0.375	0.326	0.435	0.454	0.445	0.362	0.383	0.417	0.315	0.329	0.454	0.538	0.525	0.504	0.546	0.583	0.438	0.693	1			
Q-C23	0.431	0.448	0.415	0.403	0.357	0.416	0.469	0.479	0.318	0.481	0.398	0.319	0.312	0.411	0.483	0.523	0.523	0.553	0.555	0.526	0.547	0.685	1		
Q-C24	0.476	0.587	0.475	0.464	0.353	0.462	0.582	0.574	0.461	0.446	0.463	0.308	0.228	0.563	0.487	0.593	0.596	0.655	0.499	0.619	0.472	0.543	0.559	1	
Q-C25	0.513	0.515	0.453	0.476	0.294	0.450	0.542	0.533	0.444	0.413	0.464	0.385	0.292	0.551	0.523	0.559	0.515	0.604	0.544	0.588	0.496	0.556	0.552	0.700	1

All correlations are significant at the 0.01 level (two-tailed).

### Regression analysis for quality of university’s services and university students’ satisfaction

#### Quality of university’s services and university students’ satisfaction with university social responsibility aspects

A multiple linear regression analysis was performed to predict QUS and USS based on the four aspects of USR. These variables statistically significantly predicted QUS [*F*(4.345) = 14.709, *p* < 0.000], with an *R*^2^ value of 0.146, and USS [*F*(4.345) = 13.534, *p* < 0.000], with an *R*^2^ value of 0.136. The model, which included all four aspects of USR, explained 13.6% of the variance in QUS and 12.6% of the variance in USS. Of these four variables, legal responsibilities made the largest unique contribution to QUS [beta (β) = 35], followed by that to USS [beta (β) = 31]. The remaining variables were not significantly related to the students’ overall perceptions of QUS and USS contributions to the four USR aspects. The findings are presented in Table 6.

**TABLE 6:** Multiple linear regression analysis for QUS and USS.

Variable	*t*	*P*	β	*F*	*df*	*P*	adj. *R*^2^
**QUS**							
Overall model				14.709	4	0.000**	0.136
Operational responsibilities	−0.22	0.827	−0.02				
Legal responsibilities	3.69	0.000	0.35				
Voluntary responsibilities	0.56	0.579	0.05				
Community responsibilities	0.18	0.856	0.01				
**USS**							
Overall model				13.534	4	0.000**	0.126
Operational responsibilities	0.65	0.513	0.07				
Legal responsibilities	3.20	0.001	0.31				
Voluntary responsibilities	−0.25	0.801	−0.02				
Community responsibilities	0.26	0.797	0.03				

Constant = 2.041; **p < 0.001.

#### Quality of university’s services with university students’ satisfaction

This section illustrates the results obtained from a simple linear regression analysis conducted to predict USS based on QUS. QUS statistically significantly predicted USS [*F*(1,348) = 206.134, *p* < 0.000], with an *R*^2^ value of 0.372. The model that included QUS explained 37% of the variance in USS. The findings are presented in Table 7.

**TABLE 7:** Simple regression analysis by QUS.

Variable	*t*	*P*	β	*F*	*df*	*P*	adj. *R*^2^
USS							
Overall model				205.134	1	0.000**	0.370
QUS	14.357	0.000	0.61				

Constant = 0.644; **p < 0.001.

## Discussion

To understand the nature of USR’s influence, which corresponds to the societal changes occurring in Saudi Arabia, rethinking is required in relation to the requirements of these responsibilities to meet the needs of the stakeholders and how this can be achieved in reality. In this section, the results will be discussed in relation to the nature of USR’s influence in Saudi Arabia from the perspective of the main stakeholders (i.e., the university students). Then, the mechanism of implementing USR in reality will be discussed utilizing the proposed theoretical framework: I–O map of USR.

### Students’ perceptions of university social responsibility’s aspects

Since universities must uphold their social responsibilities toward improving and developing their educational planning and strategies, there will be effects on society and the most important stakeholders (i.e., university students) (Sánchez-Hernández and Mainardes, 2016; Vázquez et al., 2016). The perceptions of university students in the current study showed a moderate to low level of agreement for fulfillment of USR in their university. This indicates the application of a low level of USR by the university and the need to fulfill these responsibilities to satisfy the students and investigate the underlying reasons for this level of agreement.

One of these reasons is the need for renovation and innovation in the scientific and practical methods and the strategies of universities to assist individual students and facilitate the economic and social construction of the society (Al-Ahmadi, 2016). Meeting the requirements of the fourth industrial revolution in the twenty-first century has become an essential ground for Saudi universities to blossom (Ismail et al., 2016; Sharabi and Faraj, 2021). Saudi Vision 2030 requires universities to build and contribute to sustainable development by exploring and preparing innovators, entrepreneurs, and decision makers to be consistent with the ambitions of Saudi society (Saudi Vision 2030, 2016).

The results of the current study showed that student satisfaction was at moderate level, while the result of the regression analysis revealed that all USR aspects explained only 13% of student satisfaction. This means that increasing USR fulfillment by the university would not affect Saudi student satisfaction significantly, suggesting the existence of other factors influencing satisfaction and student perceptions of USR.

Saudi universities have to change their learning styles and programs to prepare their students to keep up with the needs of the new labor market (Aamir et al., 2014). Ensuring that university outcomes meet the requirements of the new market is a major social responsibility of universities to benefit their graduated students, who are expected to play their roles in society (Chen et al., 2015; Buffel et al., 2017; Lo et al., 2017). The era of virtual reality and artificial intelligence as incubators for scientific and applied research imposes development in the programs, courses, and teaching methods offered by Saudi universities (Al-Ahmadi, 2016). This enables students to be consistent with upcoming changes and challenges in their lives.

Under Saudi Vision 2030, numerous vision realization programs, such as the National Transformation Program (NTP) and Human Capability Development Program (HCDP), have been launched (Saudi Vision 2030, 2021). These programs support the transformation in Saudi universities. NTP aims to develop the necessary infrastructure by supporting digital transformation, promoting social development, and ensuring the sustainability of vital resources. In contrast, HCDP aims to ensure that citizens have the required capabilities and upskilling to compete globally on the basis of a strong educational base and lifelong learning opportunities that support innovation and entrepreneurship culture (Saudi Vision 2030, 2021).

These programs can improve the quality of academic research and patents in Saudi universities, which were funded by 350 million SAR allocated for helping them to build a high-quality research strategy and identity (Ministry of Education, 2021). By 2020, the target was to publish 18,000 research papers; however, the number of published research papers reached 33.6 thousand, which shows an increase of 120%. Meanwhile, the number of registered patents in Saudi universities also increased, achieving 143 patents at local and international levels. In addition, the rank of Saudi Arabia’s social capital increased from 73rd in 2016 to 35th in 2020 (Saudi Vision 2030, 2020).

Developing and activating policies and enablers through NTP and HCDP are important to ensure Saudi universities’ competitiveness. These changes contribute to instilling values and preparing students for the future local and global labor market, achieving the students’ satisfaction toward their universities. From this perspective, the results of the current study confirmed the existence of a strong correlation between QUS and USS, while the results of regression analysis showed that, among the four USR aspects, legal responsibilities had the highest influence on students’ satisfaction.

The previous results explained the importance of the Saudi university law announced in 2019 (Saudi Universities Law, 2019), which was enforced in response to the modern direction of Saudi Vision 2030. It introduces a new mechanism that allows universities to manage their resources effectively and provides them a great opportunity to enhance their scientific, research, and community statuses at the local and international levels. The new law focuses on the role of quality in improving the outcomes of universities, which are obliged to obtain institutional and program accreditation and committed to verifying the selection of qualified staff members to manage universities’ joints efficiently (Saudi Universities Law, 2019).

Furthermore, the results of the current study displayed gender differences in favor of female students’ perceptions toward USR fulfillment by their university. This result can be explained by the ongoing changes that have been occurring for Saudi women in light of Saudi Vision 2030, leading universities to activate their roles in preparing female students to engage in the new market (Alsharif, 2019). One of the strategic targets of Saudi Vision 2030 is to empower women and invest their energies by reducing unemployment rates among them from 11.6 to 9% and increasing the proportion of women in the labor force by 2030 (Saudi Vision 2030, 2016). A report of Vision 2030 achievements from 2016 to 2020 shows that the participation of women in the labor force increased from 19.4 to 33.2% (Saudi Vision 2030, 2020).

This increase in women’s participation is attributed to a series of changes, including increase in the proportion of women leaders in the labor market by completing training of 260 female leaders and 246 managers, supporting the growth of 16 percentage points in women-owned small and medium enterprises, from 22% in 2016 to 38% in 2020 (Saudi Vision 2030, 2020). Other reforms that have been introduced to promote women’s participation include supporting access to financial services and improving wages, jobs, and working conditions, as well as making the dismissal of pregnant women illegal and guaranteeing payment of salaries during maternity leaves. Furthermore, increasing women’s participation in the industrial sector by creating new job opportunities and expanding employment opportunities for women in the private sector contributed to promoting women’s participation between 2016 and 2020 (Saudi Vision 2030, 2020). All these changes reflect on the future career of female students and development of polices of Saudi universities.

### I–O map of university social responsibility

From the theoretical perspective, on one hand, the proposed I–O map of USR aims to help universities establish the right balance between applying and utilizing their social responsibilities. On the other hand, it helps universities explore the external and internal factors that do or do not support them in fulfilling their social responsibilities. Numerous studies have been conducted globally to identify USR factors from the perspective of various stakeholders (Junyi et al., 2011; Vázquez et al., 2014; Sánchez-Hernández and Mainardes, 2016; Latif, 2018). These studies shed light on the importance of studying USR in its comprehensive concept instead of relying on stakeholders’ perceptions.

Through the analysis of the correlations among USRs, the current study confirmed the existence of a strong correlation between operational and legal responsibilities, which represents SRIC, and between voluntary and community responsibilities, which represents SROC. In contrast, among the social responsibilities toward inside and outside communities, SRHC and SRLC exist. Investigating these high and low correlations reveals existing factors that may or may not support the universities to fulfill their USRs.

The I–O map of USR, as described in the Methodology section, divides the USR correlations into the following four quadrants: SRIC, SROC, SRHC, and SRLC.

**FIGURE 1:**
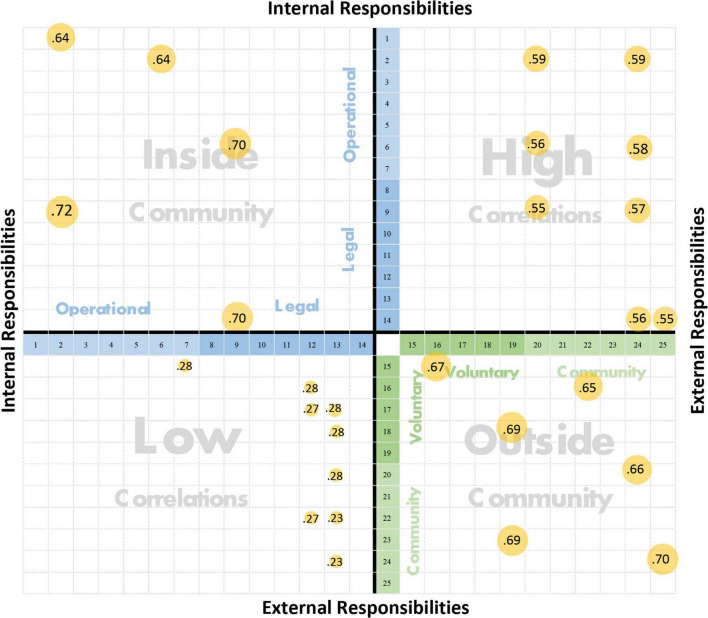
I–O map of USRs with their correlation strength scores.

The most interesting correlations among USRs with their strength scores are positioned in the I–O map of USR in each quadrant, as displayed in Figure 1.

The existence of strong correlations on the I–O map of USR among the social responsibilities toward the inside and outside community of a university asserted the nature of the university’s fulfillment of its social responsibilities as an organization (Buffel et al., 2017; Lo et al., 2017). Considering that an organization does not work in isolation from its surrounding environment, which includes its own members, society, and stakeholders (Han et al., 2020), previous studies have shown that increasing the awareness of social responsibilities’ concept for an organization is related to its influence on the inside and outside communities (Vázquez et al., 2014; Kumar, 2019).

Regarding the social responsibilities with the highest correlations, the I–O map of USR shows that a university plays the most important role in educating students about their social responsibilities in their specializations and in providing them employment opportunities. These roles correlate strongly with the university’s role for students in providing equal and diverse communication channels inside the university, ensuring the existence of a proper study environment, and respecting their rights and treating them fairly and without discrimination. From this perspective, universities apply their social responsibilities successfully toward their internal role that suits the needs of students for their expected roles in society.

In contrast, the social responsibilities with the lowest correlations on the I–O map revealed that committing to implementing regulations of behavior and activity by a university does not correlate strongly with its roles in supporting the prevention of environmental pollution, providing financial support for extracurricular activities, and supporting social and economic research projects that impact society. Likewise, the lowest correlations displayed that contributing to voluntary activities within the community and providing financial support for extracurricular activities by the university do not correlate strongly with the values, principles, and customs of the current society. This indicates the need to reformulate the regulations of universities toward serving the needs of current society, focusing on the university’s vision and expectations from community members.

Considering the weak and strong correlations, an apparent gap exists between what the university applies from its social responsibilities toward its students as internal stakeholders and how fitting that with its regulations and activities will serve the needs and changes of the surrounding environment. This is confirmed by the weakness among the social responsibilities displayed on the I–O map of USR, which shows that providing employment opportunities by universities does not correlate with the nature and reality of society. This outcome explains the necessity of the huge financial support provided by the government to the education sector (Ministry of Education, 2021), in an attempt to decrease the unemployment rates and the needs of the labor market. Thereby, the Saudi Vision 2030 is closing the gap between the community and university in Saudi Arabia by establishing many initiatives and decisions, the latest of which is the announcement of the developed Saudi universities law in 2019 (Saudi Universities Law, 2019).

## Conclusion

University social responsibility plays an important role in implementing a socially responsible management that guides societal changes and satisfies the university’s stakeholders simultaneously (Sánchez-Hernández and Mainardes, 2016). Considering that USRs internally and externally are not the same for all university stakeholders and in every society (Argandoña, 2012; Garde Sánchez et al., 2013), striking the right balance between fulfilling USR and understanding its influence on stakeholders and society simultaneously is complex. Hence, the present study aims to understand how USR helps in meeting the requirements of societal changes in Saudi Arabia from the perspective of university students by inspecting its relationship with students’ satisfaction and the quality of services offered to them. In the current study, an I–O map of USR strategy was applied to shed light on the societal factors that influence USR by investigating the correlations among USR’s aspects.

To conclude, the measurement of fulfillment of USR by a university cannot rely on the stakeholders’ opinions. Societal changes mediate the fulfillment of USR by the university and the satisfaction of its stakeholders. Therefore, universities are increasingly required to evaluate their academic purposes, visions, and corporate strategies in alignment with societal changes to ensure the effectiveness of their social responsibilities and attaining a good market reputation. Despite the lack of related studies based on the organizational structure of the university, this study attempted to utilize the available literature for building a USR scale based on measuring the perceptions of the main stakeholders (i.e., the university students) in parallel with the organizational structure of university responsibilities internally and externally simultaneously. The PCA results revealed four aspects within two dimensions: the internal responsibilities included operational and legal responsibilities and the external responsibilities included voluntary and community responsibilities. The students’ perceptions confirmed the multidimensional nature of USR perceptions under the influence of the identified societal changes, while the application of the proposed I–O map revealed how the university approaches these aspects as an organization both internally and externally.

Some of the implications of this study are related to the lack of prior scales to measure USR. Although this study proposed a USR scale as an attempt on the basis of both the organizational structure of the university, which divides the responsibilities internally and externally, and the perceptions of stakeholders in multidimensional aspects of USR, there was a lack of prior scales to measure USR on the basis of both sides. This highlights the need for ongoing research to prove its generalization to higher education and its influence on student satisfaction and the quality of services offered to them.

This study has a considerable number of limitations, mainly related to the sample of study, which consisted of university students purposefully selected from the Faculty of Social Sciences for the purpose of their awareness of USR, given that USRs are not the same for all stakeholders and in every society. Applying USR’s scales in future studies to different stakeholders and societies would facilitate the evaluation and development of these scales in different cultures. Furthermore, future studies should test the scales’ invariance and add more responsibilities to provide universities with a guideline to start.

## Data availability statement

The original contributions presented in this study are included in the article/Supplementary material, further inquiries can be directed to the corresponding author.

## Ethics statement

The studies involving human participants were reviewed and approved by the Umm Al-Qura University. The patients/participants provided their written informed consent to participate in this study.

## Author contributions

AA wrote, read, and approved the first and final drafts of the manuscript.
