# Supplementary material for: University social responsibility under the influence of societal changes: Students’ satisfaction and quality of services in Saudi Arabia
Source: Front Psychol. 2022 Sep 6;13:976192. doi: 10.3389/fpsyg.2022.976192 (PMC9487414; doi:10.3389/fpsyg.2022.976192)
Supplement: Supplementary file 1 [file Data_Sheet_1.pdf]

Supplementary Appendix 1

USR – Participants Questionnaire

Section1. Socio-demographic Information:

- Age: ☐ 19–23 ☐ 24 or More
- Gender: ☐ Man ☐ Woman

In the following statements (1–37), please express your personal opinion by selecting the most applicable answer, using the following scale of 1 (Strongly agree), 2 (Agree), 3 (Neither agree or disagree), 4 (Disagree), 5 (Strongly disagree):

Section2. Internal Social Responsibilities of University (1–14)

To what extent do you agree or disagree with your university regarding the implementation of their internal social responsibilities to fulfil the societal changes driven by Saudi Vision 2030 in the following:

2.1 Operational Responsibilities

1. Working continuously to develop condition of the educational environment	☐ 1	☐ 2	☐ 3	☐ 4	☐ 5
2. Commitment to providing equal and diverse communication channels for students	☐ 1	☐ 2	☐ 3	☐ 4	☐ 5
3. Promoting freedom of expression, dialogue, and debate	☐ 1	☐ 2	☐ 3	☐ 4	☐ 5
4. Encouraging engagement in activities that help develop knowledge, skills, and behavior	☐ 1	☐ 2	☐ 3	☐ 4	☐ 5
5. Undertaking numerous initiatives to improve the environment	☐ 1	☐ 2	☐ 3	☐ 4	☐ 5
6. Ensuring the existence of an appropriate study environment for all students	☐ 1	☐ 2	☐ 3	☐ 4	☐ 5
7. Providing all required sources of knowledge	☐ 1	☐ 2	☐ 3	☐ 4	☐ 5

2.2 Legal Responsibilities

8. Clear procedures for reporting in case of violations	☐ 1	☐ 2	☐ 3	☐ 4	☐ 5
9. Respecting student rights and treating them fairly and without discrimination	☐ 1	☐ 2	☐ 3	☐ 4	☐ 5
10. Complying with the general rules and regulations	☐ 1	☐ 2	☐ 3	☐ 4	☐ 5
11. Performing all legal duties for students	☐ 1	☐ 2	☐ 3	☐ 4	☐ 5
12. Commitment to implementing regulations of behavior and activity	☐ 1	☐ 2	☐ 3	☐ 4	☐ 5
13. Working in accordance with the values, principles, and customs of society	☐ 1	☐ 2	☐ 3	☐ 4	☐ 5
14. The existence of honesty, transparency, and integrity in all transactions	☐ 1	☐ 2	☐ 3	☐ 4	☐ 5

Section3 External Social Responsibilities of University (15–25)

To what extent do you agree or disagree with your university regarding the implementation of their external social responsibilities to fulfil the societal changes driven by Saudi Vision 2030 in the following:

3.1 Voluntary Responsibilities

15.	Contributing to voluntary activities within the community	☐ 1	☐ 2	☐ 3	☐ 4	☐ 5
16.	Undertaking steps that help prevent environmental pollution	☐ 1	☐ 2	☐ 3	☐ 4	☐ 5
17.	Providing opportunities within the community for volunteer students to expand their expertise	☐ 1	☐ 2	☐ 3	☐ 4	☐ 5
18.	Encouraging initiatives of students toward preserving the environment	☐ 1	☐ 2	☐ 3	☐ 4	☐ 5
19.	Providing financial support for extracurricular activities	☐ 1	☐ 2	☐ 3	☐ 4	☐ 5

3.2 Community Responsibilities

20.	Supporting partnerships with the private sector to develop required students' skills	☐ 1	☐ 2	☐ 3	☐ 4	☐ 5
21.	Providing university community employment opportunities	☐ 1	☐ 2	☐ 3	☐ 4	☐ 5
22.	Understanding the needs of community and working in consultation whenever possible	☐ 1	☐ 2	☐ 3	☐ 4	☐ 5
23.	Educating students about their social responsibility in their specializations	☐ 1	☐ 2	☐ 3	☐ 4	☐ 5
24.	Supporting social and economic research that impacts society	☐ 1	☐ 2	☐ 3	☐ 4	☐ 5
25.	Supporting and working with associations in line with the university's mission in society	☐ 1	☐ 2	☐ 3	☐ 4	☐ 5

Section4. Quality of University's Services (26–31)

To what extent do you agree or disagree with the quality of university services in the following statements:

26.	My university has both high-quality resources and infrastructure	☐ 1	☐ 2	☐ 3	☐ 4	☐ 5
27.	My university degree programs have a high quality	☐ 1	☐ 2	☐ 3	☐ 4	☐ 5
28.	My university's professors carry out quality tasks	☐ 1	☐ 2	☐ 3	☐ 4	☐ 5
29.	Management staff and services at my university carry out quality tasks	☐ 1	☐ 2	☐ 3	☐ 4	☐ 5
30.	My university offers quality services in comparison to others	☐ 1	☐ 2	☐ 3	☐ 4	☐ 5
31.	My university has both high-quality resources and infrastructure	☐ 1	☐ 2	☐ 3	☐ 4	☐ 5

Section5. University Students' Satisfaction (32–37)

To what extent do you agree or disagree with the following statements describing your satisfaction with the university:

32.	I am satisfied with the education offered by the university	☐ 1 ☐ 2 ☐ 3 ☐ 4 ☐ 5
33.	My decision to choose this university was correct	☐ 1 ☐ 2 ☐ 3 ☐ 4 ☐ 5
34.	I am satisfied with my overall university experience	☐ 1 ☐ 2 ☐ 3 ☐ 4 ☐ 5
35.	I will recommend this university to others	☐ 1 ☐ 2 ☐ 3 ☐ 4 ☐ 5
36.	I am proud to belong to this university	☐ 1 ☐ 2 ☐ 3 ☐ 4 ☐ 5
37.	My university experience meets my expectations	☐ 1 ☐ 2 ☐ 3 ☐ 4 ☐ 5
