# Supplementary figures and images for: University social responsibility under the influence of societal changes: Students’ satisfaction and quality of services in Saudi Arabia
Source: Front Psychol. 2022 Sep 6;13:976192. doi: 10.3389/fpsyg.2022.976192 (PMC9487414; doi:10.3389/fpsyg.2022.976192)

## Supplementary Appendix 2

## The proposed framework of I-O map of USR

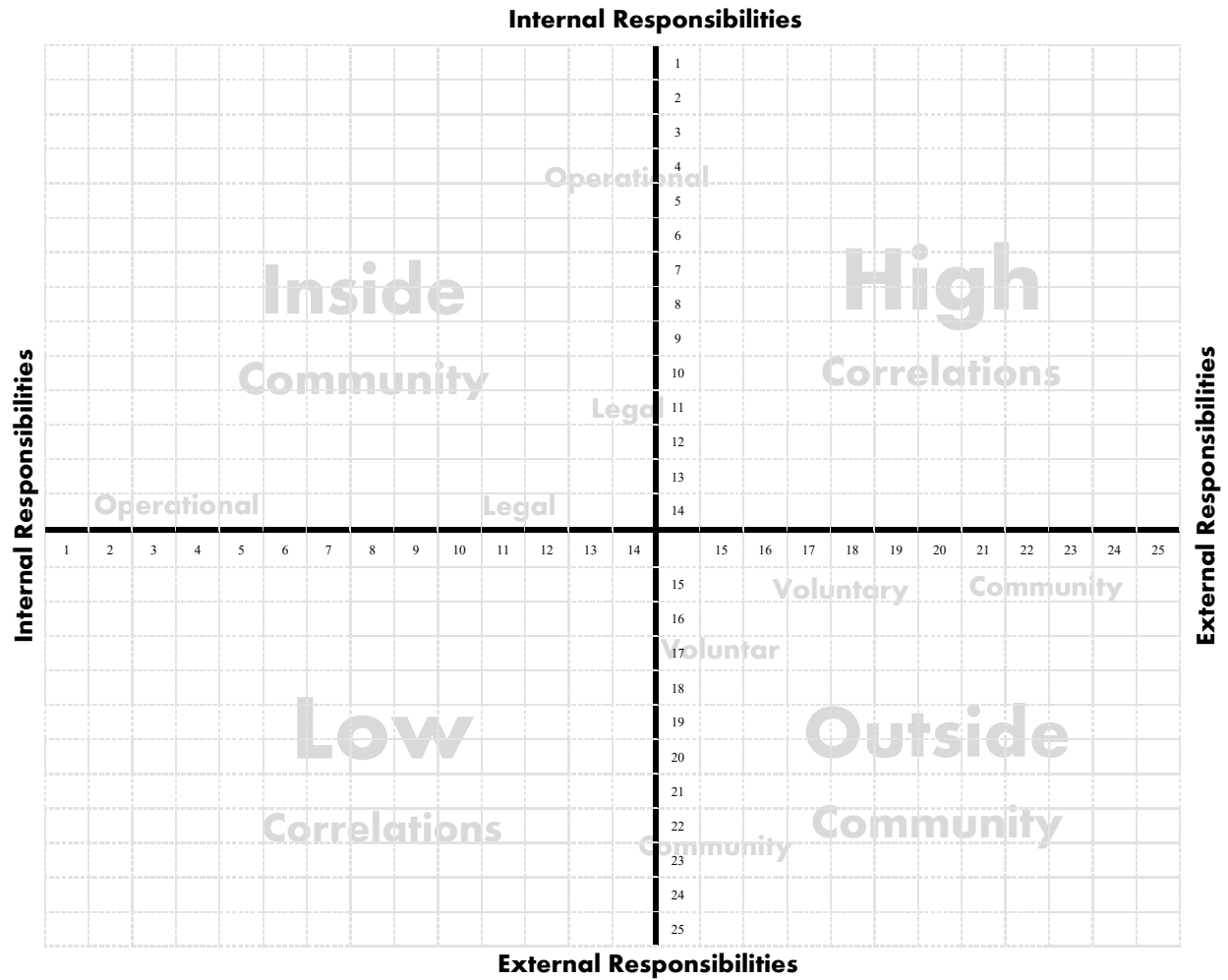

Supplement: Supplementary file 2 [file Data_Sheet_2.pdf]
