# Supplementary material for: University social responsibility under the influence of societal changes: Students’ satisfaction and quality of services in Saudi Arabia
Source: Front Psychol. 2022 Sep 6;13:976192. doi: 10.3389/fpsyg.2022.976192 (PMC9487414; doi:10.3389/fpsyg.2022.976192)
Supplement: Supplementary file 3 [file Data_Sheet_3.pdf]

Supplementary Appendix 3

Descriptive statistics for the items of each factor of USR, QUS, and USS

Factors	M	±SD
Operational Responsibilities	**1.93**	± ***.80***
- Working continuously to develop condition of the educational environment	1.82	± *.98*
- Commitment to providing equal and diverse communication channels for students	2.08	± *1.20*
- Promoting freedom of expression, dialogue, and debate	1.94	± *1.09*
- Encouraging engagement in activities that help develop knowledge, skills, and behavior	1.90	±*.94*
- Undertaking numerous initiatives to improve the environment	1.94	± *1.01*
- Ensuring the existence of an appropriate study environment for all students	1.98	± *1.17*
- Providing all required sources of knowledge	1.87	± *1.06*
Legal Responsibilities	**1.99**	± ***.85***
- Clear procedures for reporting in case of violations	2.09	± *1.15*
- Respecting student rights and treating them fairly and without discrimination	2.05	± *1.34*
- Complying with the general rules and regulations	1.97	± *1.05*
- Performing all legal duties for students	1.99	±*1.04*
- Commitment to implementing regulations of behavior and activity	1.88	± *.95*
- Working in accordance with the values, principles, and customs of society	1.72	± *.89*
- The existence of honesty, transparency, and integrity in all transactions	2.23	± *1.31*
Voluntary Responsibilities	**1.98**	± ***.79***
- Encouraging initiatives of students toward preserving the environment	1.86	± *.92*
- Undertaking steps that help prevent environmental pollution	1.99	± *1.09*
- Providing financial support for extracurricular activities	2.36	± *1.25*
- Contributing to voluntary activities within the community	1.85	±*.86*
- Providing opportunities within the community for volunteer students to expand their expertise	1.85	± *.94*
Community Responsibilities	**2.06**	± ***.88***
- Educating students about their social responsibility in their specializations	1.83	± *.99*
- Supporting partnerships with the private sector to develop required students' skills	2.25	± *1.19*
- Supporting social and economic research that impacts society	2.14	± *1.02*
- Supporting and working with associations in line with the university's mission in society	1.94	±*.98*
- Providing university community employment opportunities	2.11	± *1.30*
- Understanding the needs of community and working in consultation whenever possible	2.12	± *1.10*
QUS	**2.88**	± ***.89***
- My university has both high-quality resources and infrastructure	2.99	± *1.15*
- My university degree programs have a high quality	2.72	± *1.07*
- My university's professors carry out quality tasks	2.56	± *1.13*
- Management staff and services at my university carry out quality tasks	2.91	±*1.20*
- My university offers quality services in comparison to others	3.22	± *1.25*
- My university has both high-quality resources and infrastructure	2.99	± *1.15*
USS	**2.49**	± ***.93***
- I am satisfied with the education offered by the university	2.46	± *1.05*
- My decision to choose this university was correct	2.37	± *1.13*
- I am satisfied with my overall university experience	2.30	± *1.11*
- I will recommend this university to others	2.73	±*1.24*
- I am proud to belong to this university	2.41	± *1.15*
- My university experience meets my expectations	2.68	± *1.14*
